# Neuropathological approach to conservative treatment of scoliosis- a theoretical view point

**DOI:** 10.1186/1748-7161-7-S1-P1

**Published:** 2012-01-27

**Authors:** K Czupryna, O Nowotny-Czupryna, J Nowotny

**Affiliations:** 1 Chair of Physiotherapy in Medical University of Silesia in Katowice, Poland; 2 Department of Physiotherapy in Higher School of Administration in Bielsko-Biała, Poland

## 

Passive keeping the vertical layout is impossible because of the osteoarticular system construction, a high position of COG and the small support area. The correct layout is maintained automatically and is always introduced into the pattern encoded in the CNS. In posture regulation short back muscles play a special role.

At early development of scoliosis CNS automatically corrects irregularities, but over time habituated to them and treats them as something normal. Then we have a habit of incorrect body posture. Any attempt to restore the proper body arrangement is treated as an error and CNS automatically brings an attitude to this abnormal pattern. Later CNS treats it as a defect, which runs the compensatory mechanisms to restore the balance of the body as a whole. Then we have postural alignment, which provides a balance, but does not restore the proper body arrangement.

In the scoliosis treatment it is important to slow progression and prevent the formation of muscle imbalances and to develop the abnormal habit, which are essential part of a vicious circle, even without progression. Depending on the angle of curvature, observation, corsets and surgical treatment are recommended. A passive observation limits the possibilities of secondary prevention and interfere with the principle of rehabilitation earliness. In conservative treatment physical therapy methods are treated as unconventional treatment, questioning its effectiveness. There is no evidences that physiotherapy is not good and the sole means of secondary prevention. The biggest problem is transfer the resulting of correction for automatically adjusted corrected vertical posture [1-8].
